# Total and regional appendicular skeletal muscle mass prediction from dual-energy X-ray absorptiometry body composition models

**DOI:** 10.1038/s41598-023-29827-y

**Published:** 2023-02-14

**Authors:** Cassidy McCarthy, Grant M. Tinsley, Anja Bosy-Westphal, Manfred J. Müller, John Shepherd, Dympna Gallagher, Steven B. Heymsfield

**Affiliations:** 1 Pennington Biomedical Research Center, Louisiana State University System, 6400 Perkins Road, Baton Rouge, LA 70808 USA; 2 Department of Kinesiology and Sport Management, Texas Tech University, Lubbock, USA; 3 Department of Human Nutrition and Food Science, Christian-Albrecht’s-University of Kiel, Kiel, Germany; 4 University of Hawaii Cancer Center, Honolulu, HI USA; 5 Department of Medicine, College of Physicians and Surgeons, New York Obesity Research Center, Columbia University, New York, NY USA

**Keywords:** Medical research, Biomarkers, Diagnostic markers

## Abstract

Sarcopenia, sarcopenic obesity, frailty, and cachexia have in common skeletal muscle (SM) as a main component of their pathophysiology. The reference method for SM mass measurement is whole-body magnetic resonance imaging (MRI), although dual-energy X-ray absorptiometry (DXA) appendicular lean mass (ALM) serves as an affordable and practical SM surrogate. Empirical equations, developed on relatively small and diverse samples, are now used to predict total body SM from ALM and other covariates; prediction models for extremity SM mass are lacking. The aim of the current study was to develop and validate total body, arm, and leg SM mass prediction equations based on a large sample (N = 475) of adults evaluated with whole-body MRI and DXA for SM and ALM, respectively. Initial models were fit using ordinary least squares stepwise selection procedures; covariates beyond extremity lean mass made only small contributions to the final models that were developed using Deming regression. All three developed final models (total, arm, and leg) had high R^2^s (0.88–0.93; all p < 0.001) and small root-mean square errors (1.74, 0.41, and 0.95 kg) with no bias in the validation sample (N = 95). The new total body SM prediction model (SM = 1.12 × ALM – 0.63) showed good performance, with some bias, against previously reported DXA-ALM prediction models. These new total body and extremity SM prediction models, developed and validated in a large sample, afford an important and practical opportunity to evaluate SM mass in research and clinical settings.

## Introduction

The rising prevalence of conditions such as sarcopenia, sarcopenic obesity, frailty, and cachexia are increasingly focusing attention on the clinical measurement of skeletal muscle (SM) mass^1–5^. Many methods have been proposed for quantifying total body SM mass and the consensus is that magnetic resonance imaging (MRI) is the most applicable and safe reference method when applied in healthy adults^6^. Moreover, MRI additionally provides regional estimates of SM mass and structure^7^. However, acquiring a whole-body MRI scan is costly and automated image analysis methods are not widely available outside of proprietary vendors. Clinical studies of SM thus tend to have relatively small sample sizes, thereby limiting statistical power and generalization of results.

A widely embraced alternative to whole-body MRI for measuring muscle mass is dual-energy X-ray absorptiometry (DXA)^8,9^. A large proportion of SM is distributed in the appendages, a region in which DXA can quantify the amount of lean soft tissue present. Kim et al. in 2002 exploited this anatomic relationship and imaging capability by reporting SM mass prediction equations with DXA-measured appendicular lean soft tissue mass, now referred to as appendicular lean mass (ALM), as a key predictor variable^9^. Whole body MRI served as the reference method for measuring total SM mass in Kim’s study that included a racially/ethnically mixed model development sample of 321 adults. Kim et al. reported their findings again 2 years later^8^ following a reanalysis of 270 MRI scans that included removal of intermuscular adipose tissue (IMAT) from the SM estimates. The studies reported by Kim et al.^8,9^ did not report SM mass prediction models for regions such as the arms and legs. Studies that have followed Kim et al. identified additional SM predictor variables such as regional and total body fat mass^10,11^ on small (< 70) adult samples. Adipose tissue has a small amount of lean mass that contributes to the total ALM as measured by DXA that can lead to an overestimate of total SM mass in people with obesity^12^. Selected groups, such as young athletes, may also have ALM-SM relations that differ from those of non-athletic older adults^10^.

A series of studies over the past two decades at the Institute of Human Nutrition, Kiel University, Germany included detailed MRI total-body and regional measurements of SM and other organ and tissue volumes. Participants additionally had total-body DXA scans. The large available Kiel sample of 475 participants provides the important opportunity to develop new total body and extremity SM mass prediction equations and secondarily to validate Kim’s original total body SM prediction model^8^.

## Results

### Baseline sample characteristics

The full evaluated sample of 216 men and 259 women had a mean age of about 50 years and a BMI of 26 kg/m^2^ (Table 1). The BMI and age distributions across the model development and validation samples were similar on women and men and included wide ranges of both BMI and age. These characteristics, including MRI estimates of total body SM, are similar to those reported by Kim et al.^8^ (Supplementary Table 1).

**Table 1:** Participant Characteristics (X ± SD).

	Total sample (n = 475)		Model development (n = 380)		Model validation (n = 95)	
	Women (n = 259)	Men (n = 216)	Women (n = 212)	Men (n = 168)	Women (n = 47)	Men (n = 48)
Age (years)	49.3 ± 18.2	49.6 ± 18.1	49.7 ± 18.0	49.5 ± 18.2	47.3 ± 18.8	50.1 ± 18.2
18–39 (N)	96	73	76	58	20	15
40–59 (N)	58	63	50	48	8	15
> 60 (N)	105	80	86	62	19	18
Height (cm)	165.8 ± 6.8	178.8 ± 6.3	165.7 ± 6.9	179.0 ± 6.5	166.2 ± 6.3	178.1 ± 5.6
Weight (kg)	69.1 ± 13.0	85.1 ± 12.7	69.3 ± 13.3	84.9 ± 13.0	68.1 ± 11.7	85.6 ± 11.9
BMI (kg/m^2^)	25.1 ± 4.2	26.6 ± 3.5	25.2 ± 4.2	26.4 ± 3.4	24.6 ± 4.2	27.0 ± 3.8
UW (N)	6	2	4	2	2	0
NW (N)	144	74	116	57	28	17
OW (N)	70	104	59	83	11	21
OB (N)	39	36	33	26	6	10
MRI SM (kg)						
Total	20.0 ± 3.1	30.8 ± 4.3	19.9 ± 3.1	30.8 ± 4.5	20.1 ± 3.2	30.9 ± 3.9
Arms	2.6 ± 0.5	4.5 ± 0.8	2.6 ± 0.5	4.5 ± 0.8	2.6 ± 0.5	4.5 ± 0.7
Legs	10.1 ± 1.8	14.9 ± 2.3	10.1 ± 1.8	14.9 ± 2.3	10.2 ± 1.6	14.9 ± 2.2
ALM	12.7 ± 2.2	19.4 ± 2.9	12.7 ± 2.2	19.4 ± 3.0	12.8 ± 2.1	19.4 ± 2.8
Trunk	7.3 ± 1.2	11.4 ± 1.8	7.3 ± 1.2	11.4 ± 1.9	7.3 ± 1.5	11.5 ± 1.4
DXA lean (kg)						
Total	44.5 ± 5.9	64.3 ± 7.2	44.5 ± 6.0	64.1 ± 7.5	44.7 ± 5.8	65.1 ± 6.3
Arms	4.2 ± 0.8	7.5 ± 1.1	4.2 ± 0.8	7.5 ± 1.1	4.2 ± 0.8	7.7 ± 1.0
Legs	14.4 ± 2.5	20.4 ± 2.8	14.4 ± 2.6	20.4 ± 2.8	14.4 ± 2.3	20.6 ± 2.5
ALM	18.6 ± 3.2	28.0 ± 3.7	18.6 ± 3.3	27.9 ± 3.8	18.6 ± 2.9	28.3 ± 3.3
Trunk	24.3 ± 3.9	34.5 ± 4.6	24.3 ± 3.9	34.4 ± 4.8	24.5 ± 3.9	34.9 ± 4.2

*ALM* appendicular lean mass, *BMI* body mass index, *DXA* dual-energy X-ray absorptiometry, *MRI* magnetic resonance imaging, *N* number, *SM* skeletal muscle, *UW, NW, OW, OB* are underweight (BMI, < 18.5 kg/m^2^), normal weight (18.5–24.9 kg/m^2^), overweight (25.0–29.9 kg/m^2^), and obese (> 30 kg/m^2^).

The distribution of MRI-measured SM and DXA-measured lean mass across the total body and regions for participants in the current study is shown in Table 1. As expected, men had more total and regional SM mass than women (~ 30 vs. 20 kg). Of total SM mass, 13.0%, 36.5%, 50.5%, and 63.5% was present in the arms, trunk, legs, and appendages, respectively, in the women. Corresponding results in the men were 14.6%, 37.0%, 48.4%, and 63.0% of total body SM was present in the arms, trunk, legs, and appendages, respectively.

The proportions of DXA-measured lean mass as MRI-measured SM were largest in the legs (~ 0.70) and smallest in the trunk (~ 0.30) (Table 2); the proportion of ALM as SM was about 0.70. Men had a larger proportion of lean mass as SM in their trunk and legs and less in their arms compared to the women, although none of the regional differences were statistically significant. The proportion of total lean mass as SM was about 0.46 with the level larger in men (0.48) than in women (0.45; p = NS).

**Table 2:** Proportions of DXA-measured lean mass as MRI-measured SM (kg/kg; X ± SD).

MRI SM/ DXA lean	Total sample	Women	Men
Arms	0.61 ± 0.07	0.62 ± 0.08	0.60 ± 0.06
Legs	0.71 ± 0.06	0.70 ± 0.06	0.73 ± 0.05
Arms + legs	0.69 ± 0.05	0.68 ± 0.05	0.69 ± 0.05
Trunk	0.32 ± 0.04	0.30 ± 0.04	0.33 ± 0.04
Total body	0.46 ± 0.03	0.45 ± 0.03	0.48 ± 0.03

None of the mean differences between women and men were statistically significant.

*DXA* dual-energy X-ray absorptiometry, *MRI* magnetic resonance imaging, *SM* skeletal muscle.

### Model development and validation

The contributions of covariates on total, arm, and leg SM estimation beyond extremity lean mass components were negligible and hence the following analyses were conducted using Deming regression. The full least-squares analyses are presented in Supplementary Table 2.

### Total body

**Figure 1:**
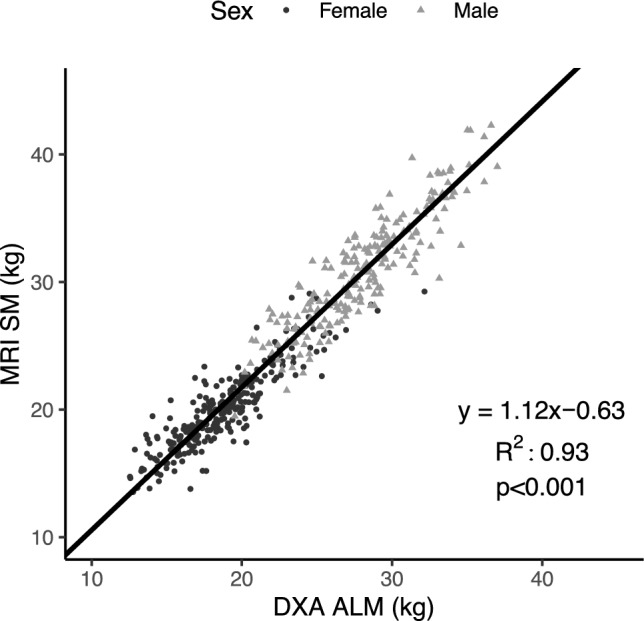
Total skeletal muscle mass (SM) measured with MRI versus appendicular lean mass (ALM) measured with DXA in the whole sample (n = 475). The Deming regression equation, line (solid), and R^2^ are shown in the figure (p < 0.001).

**Figure 2:**
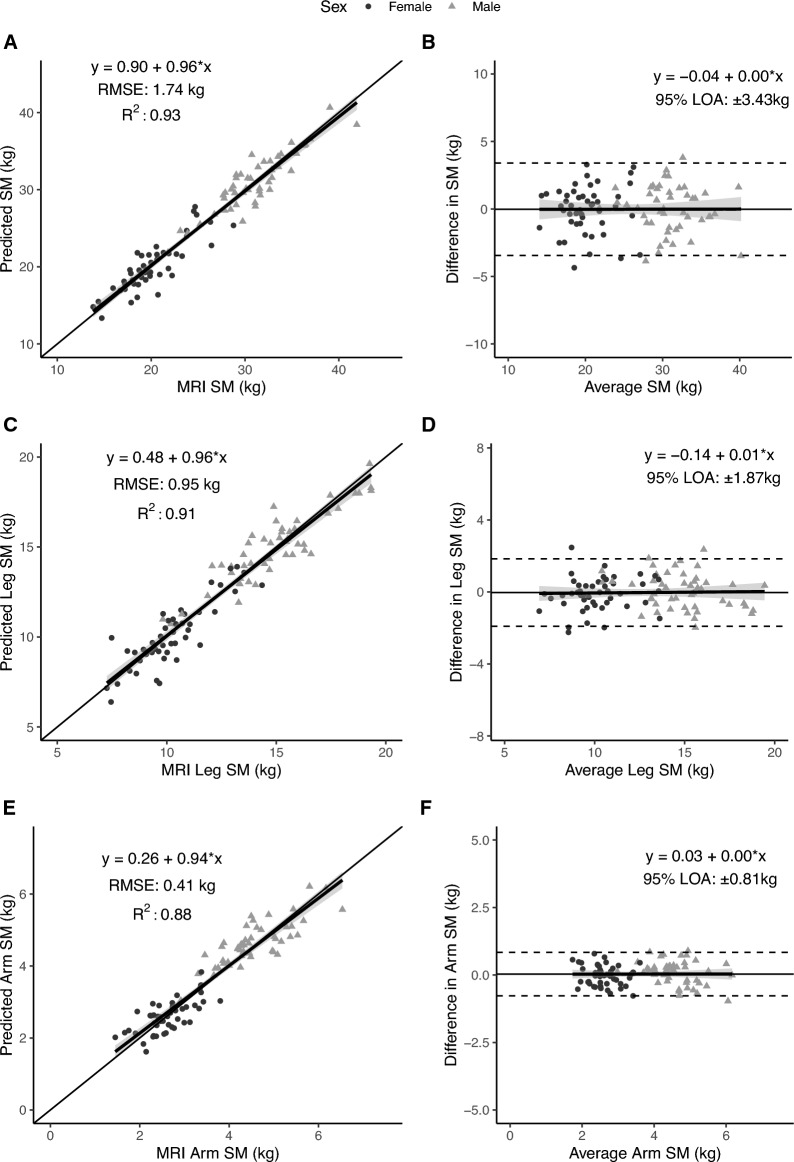
Predicted total, arm, and leg skeletal muscle (SM) mass versus corresponding value measured with MRI in the validation sample (n = 47 women; 48 men) on the left (**A,C,E**) and associated Bland–Altman plots on the right (**B,D,F**). The regression equations, lines, R^2^s, and 95% limits of agreement (LOA) are shown in the figures. The statistical significance of each panel is summarized in the text.

The strong association between MRI-measured total body SM mass and DXA-measured ALM is shown for the full sample in Fig. 1 (R^2^, 0.93; p < 0.001). The total body SM mass prediction model (Table 3, Fig. 2A) had a validation R^2^ of 0.93 and RMSE of 1.74 kg. No significant difference from the line of identity was observed for the slope (95% CI: 0.91, 1.02) and intercept (95% CI: −0.56, 2.35). Additionally, statistical equivalence was demonstrated (p < 0.001) between MRI-SM and predicted SM mass using regions of 2.5% of MRI-SM (0.64 kg). No significant proportional bias was observed in the Bland–Altman analysis (slope 95% CI: −0.06, 0.06; Fig. 2B). Sex-specific models and their performance are presented in the Supplementary Tables 3 and 4 and Supplementary Figs. 1 and 2).

**Table 3:** Developed SM mass prediction models.

Dependent Variable	Model	Equation	Slope 95% CI	Intercept 95% CI	Validation (n = 95)	
					RMSE (kg)	R^2^
Total SM	Development (n = 380)	1.12 × ALM – 0.67	1.08, 1.15	−1.51, 0.18	1.74	0.93
	Final (n = 475)	1.12 × ALM – 0.63	1.08, 1.15	−1.45, 0.19	–	–
Leg SM	Development (n = 380)	0.78 × leg lean – 1.16	0.75, 0.81	−1.63, −0.68	0.95	0.91
	Final (n = 475)	0.78 × leg lean – 1.07	0.75, 0.80	−1.50, −0.64	–	–
Arm SM	Development (n = 380)	0.58 × arm lean + 0.15	0.56, 0.60	0.03, 0.26	0.41	0.88
	Final (n = 475)	0.58 × arm lean + 0.15	0.56, 0.60	0.04, 0.26	–	–

ALM and lean mass units are in kg.

*ALM* appendicular lean mass, *RMSE* root mean square error, *SM* skeletal muscle.

### Leg

The leg SM mass prediction model (Table 3, Fig. 2C) had a validation R^2^ of 0.91 and RMSE of 0.95 kg. No significant difference from the line of identity was observed for the slope (95% CI: 0.90, 1.02) or intercept (95% CI: −0.35, 1.30). Additionally, statistical equivalence was demonstrated (p < 0.001) between MRI leg SM and predicted leg SM using regions of 5% of MRI arm SM (0.61 kg). No significant proportional bias was observed in the Bland–Altman analysis (slope 95% CI: −0.06, 0.07; Fig. 2D).

### Arm

The arm SM mass prediction model (Table 3, Fig. 2E) had a validation R^2^ of 0.88 and RMSE of 0.41 kg. No significant difference from the line of identity was observed for the slope (95% CI: 0.87, 1.01) or intercept (95% CI: −0.02, 0.53). Additionally, statistical equivalence was demonstrated (p < 0.001) between MRI arm SM and predicted arm SM mass using regions of 5% of MRI arm SM (0.17 kg). No significant proportional bias was observed in the Bland–Altman analysis (slope 95% CI: −0.07, 0.08; Fig. 2F).

### Model cross-validations

#### SM predicted by Kim equation vs. SM measured at Kiel

**Figure 3:**
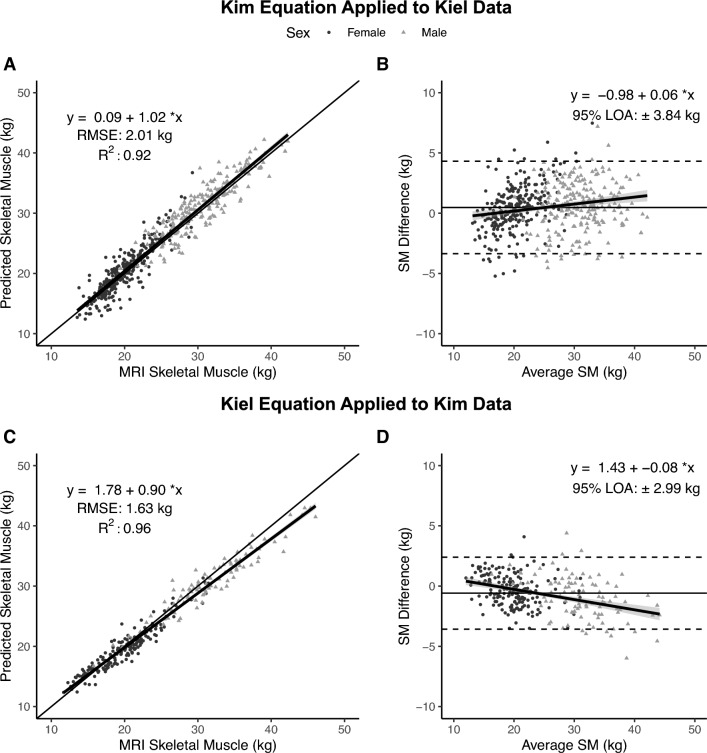
Total skeletal muscle (SM) mass predicted by Kim’s equation^8^ versus SM measured with MRI at Kiel (**A**) and corresponding Bland–Altman plot (**B**) (n = 475). Total body skeletal muscle (SM) mass predicted by the newly developed Kiel equation versus SM measured with MRI by Kim et al.^8^ (**C**) and corresponding Bland–Altman plot (**D**) (n = 270). The lines of identity (thin solid line), regression equations and lines (solid lines with gray shading indicating 95% CI), and R^2^s are shown in (**A,C**). The regression lines with 95% CI and 95% limits of agreement (LOA) (dashed lines) are shown in (**B,D**). Statistical significance of each panel is summarized in the text.

ALM measured at Kiel was used to derive a total body SM estimate using Kim’s multivariate SM prediction model^8^ (SM = 1.18 × ALM − 0.03 × Age – 0.14). The Kim-predicted SM values were then compared to MRI-measured SM at Kiel. Cross-validation of Kim’s model indicated no significant difference from identity for the line’s slope (95% CI: 0.99, 1.04) or intercept (95% CI: −0.60, 0.78) (Fig. 3A); RMSE was 2.01 kg and R^2^ 0.92. There was a trend for statistical equivalence (p = 0.051) using regions of 2.5% of MRI SM (0.62 kg). There was significant proportional bias observed (95% CI for slope: 0.03, 0.08) in the Bland–Altman plot (Fig. 3B).

#### SM predicted by Kiel equation vs. SM measured by Kim et al.^8^

ALM measured by Kim et al.^8^ was used to derive a total body SM estimate using the newly developed Kiel SM prediction model. The Kiel-predicted SM values were then compared to MRI-measured SM by Kim et al.^8^ Cross-validation of the Kiel equation indicated a significant difference from the line of identity for the slope (95% CI: 0.88, 0.92) and intercept (95% CI: 1.24, 2.32) (Fig. 3C); the RMSE was 1.63 kg with an R^2^ of 0.96. Statistical equivalence was not observed (p = 0.44) using regions of 2.5% of MRI SM (0.60 kg). Significant proportional bias was observed (95% CI for slope: −0.11, −0.06) in the Bland–Altman plot (Fig. 3D).

## Discussion

New total body and extremity SM mass prediction equations were developed and validated in the current study using MRI and DXA data from a large sample evaluated at Kiel University’s Institute of Human Nutrition. Additionally, we examined the widely used Kim SM prediction model in the current sample and cross-validated the newly developed total body SM prediction equation with Kim’s original MRI and DXA data.

Several observations emerge from the current study results and the other previously reported smaller scale and more limited studies^10,11^ aimed at developing total body SM prediction equations that are summarized in Table 4. First, we again confirmed that about 60–70% of total body SM is present in the extremities and that extremity lean mass (i.e., ALM) is about 70% SM. It’s not surprising, therefore, that total body SM and ALM are highly correlated with each other; the univariate regression R^2^ in the current study for SM versus ALM was 0.93 (p < 0.001) and in Kim’s study^8^ 0.96 (p < 0.001). Similar strong associations between SM and ALM were observed by Zhao et al. in 66 Chinese men and women (R^2^, 0.97, p < 0.001)^11^ and to a less extent by Sagayama in 30 young athletic Japanese men (r, 0.885, p < 0.001; ref 10)^10^ (Table 4). By contrast to ALM, trunk lean mass was only one-third SM, the rest presumably visceral lean tissues such as liver, kidneys, and heart. These observations thus again affirm that DXA ALM is an excellent starting point for developing total body SM prediction equations.

**Table 4:** Comparison of previously reported and current SM prediction models.

Study	Sample N (M/W)	DXA scanner	Model
Current study	475 (216/259) Caucasian adults; mean age ~ 50 years; BMI, ~ 26 kg/m^2^	QDR 4500A, Hologic	1.12 × ALM – 0.63
Kim et al.^8^	270 racially/ethnically mixed adults; mean age ~ 46 years; BMI, ~ 25 kg/m^2^	DPX, Lunar	SM = 1.19 × ALM – 1.65 SM = 1.18 × ALM − 0.03 × age – 0.14
Sagayama et al.^10^	30 Japanese athletic men; mean age 19.9 years; BMI, 23.7 kg/m^2^	Discovery A, Hologic	SM = 1.21 × ALM + 21.85 × trunk/ALM—0.35 × %fat – 18.41
Zhao et al.^11^	66 (52/14) Chinese adults; mean age ~ 50 years; BMI, ~ 23 kg/m^2^	Prodigy, GE Lunar	SM = 1.21 × ALM – 0.98 age < 45 years SM = 1.21 × ALM − 0.98 – 0.04 × (age – 45) age ≥ 45 years

Mass units, kg; Sex, 0, woman; 1, man.

*A* age, *ALM* appendicular lean mass, *BMI* body mass index, *M* men, *SM* skeletal muscle, *W* women.

Previous studies have included additional covariates beyond ALM in SM prediction models. Specifically, for the total body SM prediction models, age, sex, muscle distribution, and %fat appear as covariates in earlier prediction equations shown in Table 4. These observations may be expected as the developed SM prediction models are empirical and capture associations that likely reflect small variations in SM distribution as a function of sex, age, athletic “fitness”, and variation in non-muscle lean mass composition^13^. However, in the present analysis, the contribution of such covariates on SM estimation was negligible (Supplementary Table 2). As such, a simplified model using ALM as the sole predictor of SM and accounting for errors in both MRI and DXA estimates was developed. Similarly, the developed leg and arm SM estimation equations use solely leg LM and arm LM as predictors, respectively. The current study sample included only Caucasian participants and we could therefore not establish if significant race or ethnicity covariates might enter the SM prediction model as reported by Kim et al.^8^ Collectively, ALM and extremity lean are the main predictors of total and regional SM, respectively, which allows for the development and utilization of parsimonious prediction models. However, small contributions to between-individual variance can be made by other covariates in some instances.

In the current study we also observed that SM predicted by Kim’s model and the models reported by Zhao et al.^11^ and Sagayama et al.^10^ did not predict identical values to those estimated from our new total body SM model. We found small absolute differences and some bias in predicted SM between our model and Kim’s model,^8^ even when using Kim’s original data. Moreover, we similarly found strong correlations but absolute differences and bias when applying Zhao and Sagayama’s models^10,11^ to the Kiel dataset (data not shown). These kinds of SM prediction variation can be anticipated due to between-sample, DXA system^14^, MRI scanner, and image segmentation method differences (Table 4). To explore the magnitude of potential DXA scanner differences, we compared Hologic Discovery and GE Lunar iDXA estimates of ALM in our laboratory (Supplementary Fig. 3), similar to the study reported by Park et al.^14^ that compared Hologic Horizon and GE Lunar Prodigy scanners. Although ALM measured by both DXA systems in our laboratory were highly correlated (n = 45; R^2^, 0.99), average scanner differences (X ± SD; 0.54 ± 0.58 kg; p < 0.001) and significant bias (p < 0.05) were present. Park et al.^14^ found between-scanner ALM differences of 1.79 ± 0.92 kg (p < 0.001). These kinds of between DXA and MRI system measurement differences are likely part of the reason why we found small mean differences and bias between our SM predictions and those of Kim et al.^8^

A concern raised in several previous publications is that DXA ALM, and thus predicted SM, is not “true” muscle mass^15^. As noted earlier, ALM is linked to SM through several additional covariates. Thus, for example, when people with obesity lose weight some of the changes observed in ALM may be accounted for by changes in the lean portion of appendicular adipose tissue. Preferential changes in SM distribution with interventions might also impact predictions with empirical total body SM equations. Lastly, DXA-predicted SM and SM measured with MRI share in common an evaluation of “total” wet muscle that includes tendons, nerves, blood vessels, and connective tissues. Other methods, such as D_3_-creatine dilution^15^, multifrequency bioimpedance analysis^16^, and ultrasound^17^ can be used to derive estimates of muscle “quality” that go beyond an evaluation of the total intact muscle.

Although the current study models were developed on the largest sample to date, ideally much larger and more diverse samples should be evaluated in the future. This limitation will likely be overcome when automated MRI analysis software becomes available, thus reducing study analysis cost and execution time. Our study participants were all Caucasian, and thus generalizing our SM prediction models to other race and ethnic groups optimally should include a priori validation. Our models also did not include potential covariates such as fitness level or type and duration of exercise training. Participants in the current study were also healthy and fully functional and thus a need exists to expand model development samples to people at the extremes of muscularity such as patients with conditions such as sarcopenia on the one hand and body builders on the other. Lastly, we limited our participants to those with BMIs < 35 kg/m^2^. While it is feasible to conduct DXA and MRI scans in people with higher BMIs, measurement errors increase at and above this level of adiposity and thus we elected, as did Kim et al.^8^, to stay with BMIs < 35 kg/m^2^.

The current study advanced a new set of total body and extremity SM prediction equations that should be useful in the study of conditions related to variation in muscularity. Even the relatively small arm lean component yielded a good arm SM prediction equation with a small RMSE of 0.41 kg. Our models are founded on a large sample of healthy adults ranging in age and BMI. The current study findings also suggest the need to standardize DXA and MRI measurement methods and analyses across centers with the aim of creating universal DXA SM prediction models. Combining predicted values for total SM with other estimates of muscle “quality” provides an important opportunity for future research.

## Methods

### Study design

The primary aim of this study was to develop and validate new total body and regional DXA SM prediction equations. Each participant had a whole-body MRI and DXA scan on the same day as reported in previous protocols^18–21^. As in the study of Kim et al.^8^, IMAT-free SM prediction models were developed for participants whose body mass index (BMI) was < 35 kg/m^2^. The initial study phase involved development of SM mass prediction equations for the total body, arms, and legs in a portion of the sample; a validation phase in the remaining sample followed. The Kiel University Institutional Review Board approved the involved studies and participants signed informed consents before commencing the evaluation protocols. All research was performed in accordance with relevant guidelines/regulations including the Declaration of Helsinki.

The secondary aim of the study was to validate Kim’s original total body SM prediction model^8^, one that was applicable to the current study. We conducted these analyses in two stages. First, we used Kim’s total body SM prediction model, as specified below, to derive a predicted SM value for each participant in the current study. These SM estimates were then compared to the actual SM values derived with MRI in the current study. The second step was to compare our newly derived total body SM prediction model to SM estimated with MRI in Kim’s original study^8^. The raw SM data from the study reported by Kim et al.^8^ was available to the current investigators.

### Participants

Participants in the Kiel sample were healthy ambulatory Caucasian men and women, age ≥ 18 years, who were engaged in regular physical activities but not in programmed exercise training programs or in sports competitions. The allocation of participants in the full Kiel sample to the current study is outlined in Supplementary Fig. 4. Of 548 total available participants, 475 were evaluated in this study who had BMIs of < 35 kg/m^2^. The sample reported by Kim et al.^8^ included 270 adults (96 men, 174 women) with demographic characteristics shown in Supplementary Table 1.

### Measurements

The deep phenotyping protocol at the Kiel Institute of Human Nutrition is reported in detail in earlier publications^12,18–21^. Body weight and height were measured in each participant ± 0.01 kg with a digital scale (Tanita Corp., Tokyo, Japan) and ± 0.5 cm with a mechanical stadiometer (Seca, Hamburg, Germany), respectively.

A 1.5 T Magneton Vision or Avanto Siemens scanner (Siemens Medical Systems, Erlangen, Germany) was used to quantify total body (wrist-to-ankle) and regional (arms, legs, trunk) skeletal muscle volumes that were converted to mass assuming a muscle density of 1.04 kg/l. The generated cross-sectional images were manually analyzed by a skilled technician with SliceOmatic software (version 4.3, Tomovision, Montreal, Canada).

A whole-body DXA scanner (QDR 4500A, Hologic, Marlborough, Massachusetts) that operated with software version V8.26a:3.19 was used to evaluate total body and regional (arms, legs, trunk) fat, lean mass, and bone mineral content; ALM was calculated as the sum of lean mass present in both arms and legs. System calibrations were conducted on a regular basis as specified by the manufacturer.

A 1.5-T 6X Horizon MRI scanner (General Electric, Milwaukee, WI) and a Lunar DPX (software version 3.6, GE Healthcare, Madison, WI) were used to quantify SM and ALM, respectively, in Kim’s studies. Details of the MRI and DXA acquisition protocols are reported in Kim et al.^8,9^

### Statistical methods

From the full evaluated dataset of 475 participants (Supplementary Fig. 4), 80% were randomly selected for use in initial model development (n = 380; 212 women, 168 men) with the remaining 20% of participants used as the validation dataset (n = 95; 47 women, 48 men). Preliminary models were fit using ordinary least squares linear regression procedures with stepwise selection. Potential predictor variables included DXA lean mass (arms, legs, and ALM), sex, age, and DXA fat mass (arms, legs, and total appendicular). Using tenfold cross-validation, the prediction error was estimated through the root mean square error (RMSE) and coefficient of determination (R^2^). Based on evaluation of the RMSE and R^2^ of candidate models, as well as subsequent validation performance of models, it was determined that no variables beyond lean mass (i.e., ALM, arm lean mass, or leg lean mass) exerted a meaningful influence on skeletal muscle estimation models (Supplementary Table 2). Therefore, parsimonious models with a single LM predictor were developed. As a single predictor variable was to be used in each regression equation, models were fit using Deming regression, which accounts for error in the measurement of both the predictor and outcome variables (i.e., DXA LM and MRI SM estimates). These models were initially fit using the model development group (n = 380) to allow for model validation using the separate validation dataset (n = 95). For validation, skeletal muscle mass predicted from the developed equations was compared to MRI-measured SM mass through a comparison with the line of identity (i.e., the perfect relationship between values, with a slope of 1 and intercept of 0), calculation of the RMSE and R^2^, Bland–Altman analysis, and equivalence testing. For equivalence testing, equivalence regions were established as 2.5% of total-body SM and 5% of arm and leg SM. These regions were selected based on prior work utilizing equivalence regions of 5% for total fat-free mass^22^, the expectation of higher relative errors for regional estimates, and the desire to employ a conservative equivalence region for total-body SM. After validation, final models were fit using the entire sample (n = 475), with negligible differences between the coefficients in the development and final models (Table 3).

Despite the negligible impact of sex and age terms within the SM prediction models, additional equations were developed within men only and women only to allow use of these equations in situations where an equation developed within a single gender is preferable (Supplementary Tables 3 and 4 and Supplementary Figs. 1 and 2). The total body SM prediction equation of Kim et al. (SM (kg) = 1.18 × ALM (kg) −0.03 × age −0.14)^8^ was used to derive SM estimates using Kiel’s DXA-measured ALM and age of the full sample. Predicted and measured SM were then compared using regression and Bland–Altman analyses. The same analysis approach was used to compare SM predicted using our newly developed total body SM model to Kim’s MRI-measured SM; ALM and age values in Kim’s sample were used as model covariates. Statistical significance was accepted at p < 0.05. Sample descriptive statistics are reported as the mean ± SD. Data analysis was performed using R software (v. 4.2.1)^23^ with the packages caret (v. 6.0–93)^24^, TOSTER (v. 0.4.2)^25^, DescTools (v. 0.99.46)^26^, and Deming (v. 1.4)^26^.

### Ethical approval

The Kiel University Institutional Review Board approved the involved studies and participants signed informed consents before commencing the evaluation protocols.

### Supplementary Information

Supplementary Information.

## Data Availability

Data described in this manuscript will be made available upon request and approval by the principal investigator, Steven B. Heymsfield (steven.heymsfield@pbrc.edu).

## Abbreviations

ALM: Appendicular lean mass
BMI: Body mass index
CV: Coefficient of variation
DXA: Dual-energy X-ray absorptiometry
IMAT: Intermuscular-adipose tissue
LOA: Limits of agreement
MRI: Magnetic resonance imaging
PBRC: Pennington Biomedical Research Center
RMSE: Root mean square error
