# Publisher Correction: Total and regional appendicular skeletal muscle mass prediction from dual-energy X-ray absorptiometry body composition models

**DOI:** 10.1038/s41598-023-39896-8

**Published:** 2023-08-09

**Authors:** Cassidy McCarthy, Grant M. Tinsley, Anja Bosy-Westphal, Manfred J. Müller, John Shepherd, Dympna Gallagher, Steven B. Heymsfield

**Affiliations:** 1 Pennington Biomedical Research Center, Louisiana State University System, 6400 Perkins Road, Baton Rouge, LA 70808 USA; 2 Department of Kinesiology and Sport Management, Texas Tech University, Lubbock, USA; 3 Department of Human Nutrition and Food Science, Christian-Albrecht’s-University of Kiel, Kiel, Germany; 4 University of Hawaii Cancer Center, Honolulu, HI USA; 5 Department of Medicine, College of Physicians and Surgeons, New York Obesity Research Center, Columbia University, New York, NY USA

Correction to: *Scientific Reports* https://doi.org/10.1038/s41598-023-29827-y, published online 14 February 2023

The original version of this Article contained errors.

In Figures 2 and 3, the labels and text did not display correctly.

The original Figures 2 and 3 and accompanying legends appear below.

**Figure 2:**
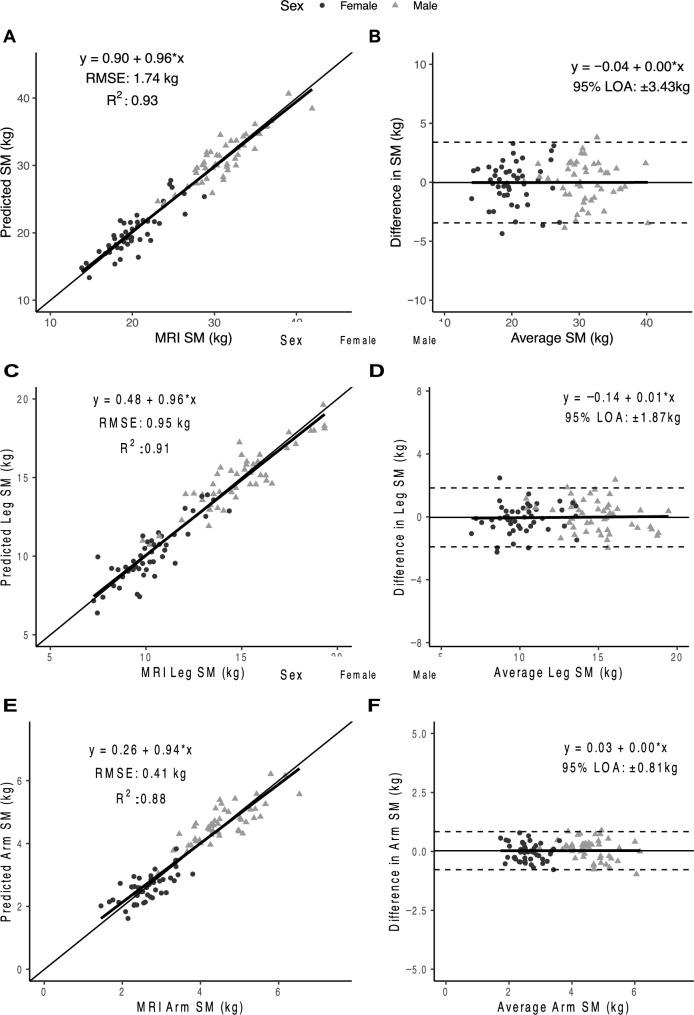
Predicted total, arm, and leg skeletal muscle (SM) mass versus corresponding value measured with MRI in the validation sample (n = 47 women; 48 men) on the left (**A**,**C**,**E**) and associated Bland–Altman plots on the right (**B**,**D**,**F**). The regression equations, lines, R^2^s, and 95% limits of agreement (LOA) are shown in the figures. The statistical significance of each panel is summarized in the text.

**Figure 3:**
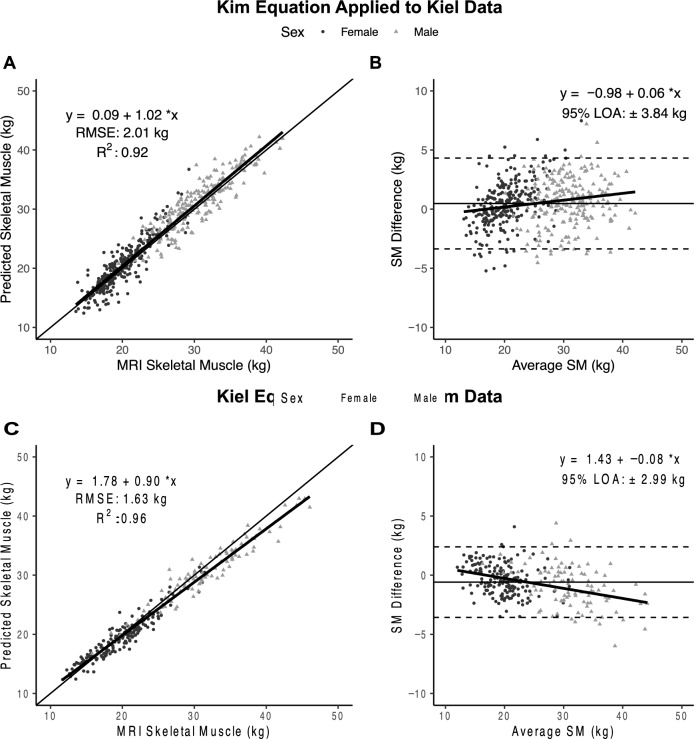
Total skeletal muscle (SM) mass predicted by Kim’s equation^8^ versus SM measured with MRI at Kiel (**A**) and corresponding Bland–Altman plot (**B**) (n = 475). Total body skeletal muscle (SM) mass predicted by the newly developed Kiel equation versus SM measured with MRI by Kim et al.^8^ (**C**) and corresponding Bland–Altman plot (**D**) (n = 270). The lines of identity (thin solid line), regression equations and lines (solid lines with gray shading indicating 95% CI), and R^2^s are shown in (**A**,**C**). The regression lines with 95% CI and 95% limits of agreement (LOA) (dashed lines) are shown in (**B**,**D**). Statistical significance of each panel is summarized in the text.

The original Article has been corrected.

