# Supplementary material for: Total and regional appendicular skeletal muscle mass prediction from dual-energy X-ray absorptiometry body composition models
Source: Sci Rep. 2023 Feb 14;13:2590. doi: 10.1038/s41598-023-29827-y (PMC9929067; doi:10.1038/s41598-023-29827-y)
Supplement: Supplementary file 1 — Supplementary Information. [file 41598_2023_29827_MOESM1_ESM.docx]

**SUPPLEMENTARY MATERIAL**

**Total and Regional Appendicular Skeletal Muscle Mass: Prediction from Dual-Energy X-ray Absorptiometry Body Composition Models**

Cassidy McCarthy^1^, Grant M. Tinsley^2^, Anja Bosy-Westphal^3^, Manfred J. Muller^3^, John Shepherd^4^, Dympna Gallagher^5^, Steven B. Heymsfield^1^

^1^Pennington Biomedical Research Center, Louisiana State University System, Baton Rouge, LA, USA; ^2^Department of Kinesiology & Sport Management, Texas Tech University; ^3^Department of Human Nutrition and Food Science, Christian-Albrecht’s-University of Kiel, Kiel,

Germany; ^4^University of Hawaii Cancer Center, Honolulu, HI, USA; ^5^New York Obesity Research Center, Department of Medicine, College of Physicians and Surgeons, Columbia University, New York, NY, USA.

**Address Correspondence to:**Steven B. Heymsfield, MD
Pennington Biomedical Research Center
6400 Perkins Road
Baton Rouge, LA, USA 70808

**Tel**: 225-763-2541

**Fax**: 225-763-3030

**E-mail**: [steven.heymsfield@pbrc.edu](mailto:steven.heymsfield@pbrc.edu)

**Contents
Table 1.** Kim study participant characteristics
**Table 2.** Initial stepwise selection regression models for skeletal muscle estimation

**Table 3.** Developed SM mass prediction models in males only

**Table 4.** Developed SM mass prediction models in females only

**Figure 1.** Predicted skeletal muscle validation in males

**Figure 2.** Predicted skeletal muscle validation in females

**Figure 3.** Appendicular lean mass (ALM) measured by Discovery/Horizon W and
 iDXA/Prodigy scanners
**Figure 4.** Consort diagram showing participant samples

**Table 1**. Kim’s study participant characteristics (X±SD)^1^.

|  | Total Sample (n=270) | |
| --- | --- | --- |
|  | **Women** (n= 174) | **Men** (n= 96) |
| Age (y) | 45.2 ± 17.6 | 46.4 ± 19.0 |
| Height (cm) | 162.3 ± 7.5 | 176.4 ± 7.9 |
| Weight (kg) | 64.5 ± 13.6 | 79.2 ± 12.2 |
| BMI (kg/m^2^) | 24.4 ± 4.4 | 25.4 ± 3.0 |
| MRI-SM (kg) | 19.8 ± 3.9 | 31.7 ± 5.9 |
| DXA-ALM (kg) | 18.0 ± 3.2 | 28.1 ± 4.6 |

Values are X ± SD. Abbreviations: ALM, appendicular lean mass; BMI, body mass index; DXA, dual-energy X-ray absorptiometry; MRI, magnetic resonance imaging; SM, skeletal muscle.

1. Kim J, Heshka S, Gallagher D, Kotler DP, Mayer L, Albu J, Shen W, Freda PU, Heymsfield SB. Intermuscular adipose tissue-free skeletal muscle mass: estimation by dual-energy X-ray absorptiometry in adults. J Appl Physiol. 2004;97:655-60. doi: 10.1152/japplphysiol.00260.2004. Epub 2004 Apr 16. PMID: 15090482.

**Table 2.** Initial stepwise selection regression models for skeletal muscle estimation.

|  | Predictors | RMSE | RMSE SD |  | R^2^ | R^2^ SD |  | MAE | MAE SD |
| --- | --- | --- | --- | --- | --- | --- | --- | --- | --- |
| Total SM | ALM | 1.80 | 0.14 |  | 0.93 | 0.02 |  | 1.38 | 0.11 |
|  | ALM, Sex | 1.70 | 0.22 |  | 0.94 | 0.02 |  | 1.32 | 0.14 |
|  | ALM, Sex, Age | 1.70 | 0.23 |  | 0.94 | 0.02 |  | 1.31 | 0.14 |
| Leg SM | Leg LM | 0.93 | 0.15 |  | 0.91 | 0.03 |  | 0.71 | 0.13 |
|  | Leg LM, Sex | 0.91 | 0.14 |  | 0.92 | 0.03 |  | 0.70 | 0.12 |
|  | Leg LM, Sex, Age | 0.87 | 0.15 |  | 0.92 | 0.03 |  | 0.65 | 0.13 |
| Arm SM | Arm LM | 0.40 | 0.04 |  | 0.88 | 0.02 |  | 0.32 | 0.04 |
|  | Sex, Age | 0.55 | 0.07 |  | 0.77 | 0.05 |  | 0.42 | 0.05 |
|  | Arm LM, Sex, Age | 0.37 | 0.04 |  | 0.90 | 0.02 |  | 0.29 | 0.04 |

10-fold cross-validation values are displayed. RMSE: root mean square error; MAE: mean absolute error.

**Table 3**. Developed SM mass prediction models in males only.

|  |  |  |  |  | Validation (n=95) | |
| --- | --- | --- | --- | --- | --- | --- |
| Dependent Variable | Model | Equation | Slope  95% CI | Intercept  95% CI | RMSE (kg) | R^2^ |
| Total SM | Development (n=168) | 1.18 x ALM – 2.23 | 1.09, 1.28 | -4.90, 0.43 | 1.80 | 0.80 |
|  | Final (n=216) | 1.18 x ALM – 2.13 | 1.10, 1.26 | -4.40, 0.14 | - | - |
| Leg SM | Development (n=168) | 0.83 x leg lean – 2.02 | 0.77, 0.89 | -3.23, -0.81 | 0.99 | 0.81 |
|  | Final (n=216) | 0.84x leg lean – 2.36 | 0.79, 0.90 | -3.41, -1.31 | - | - |
| Arm SM | Development (n=168) | 0.70 x arm lean – 0.75 | 0.62, 0.79 | -1.38, -0.13 | 0.47 | 0.65 |
|  | Final (n=216) | 0.70 x arm lean – 0.78 | 0.63, 0.77 | -1.33, -0.24 | - | - |

ALM and lean mass units are in kg. Abbreviations: ALM, appendicular lean mass; RMSE, root mean square error; SM, skeletal muscle.

**Table 4**. Developed SM mass prediction models in females only.

|  |  |  |  |  | Validation (n=95) | |
| --- | --- | --- | --- | --- | --- | --- |
| Dependent Variable | Model | Equation | Slope  95% CI | Intercept  95% CI | RMSE (kg) | R^2^ |
| Total SM | Development (n=212) | 0.96 x ALM + 1.98 | 0.89, 1.03 | 0.66, 3.30 | 1.69 | 0.72 |
|  | Final (n=259) | 0.98 x ALM + 1.63 | 0.91, 1.05 | 0.36, 2.91 | - | - |
| Leg SM | Development (n=212) | 0.73 x leg lean – 0.43 | 0.67, 0.78 | -1.22, 0.36 | 0.89 | 0.74 |
|  | Final (n=259) | 0.73x leg lean – 0.38 | 0.67, 0.78 | -1.35, 0.60 | - | - |
| Arm SM | Development (n=212) | 0.57 x arm lean + 0.19 | 0.50, 0.64 | -0.09, 0.46 | 0.37 | 0.50 |
|  | Final (n=259) | 0.59 x arm lean + 0.10 | 0.56, 0.60 | -0.22, 0.43 | - | - |

ALM and lean mass units are in kg. Abbreviations: ALM, appendicular lean mass; RMSE, root mean square error; SM, skeletal muscle.

**Figure 1.** Predicted skeletal muscle validation in males

**Figure 2.** Predicted skeletal muscle validation in females

**Figure 3.** Appendicular lean mass (ALM) measured by Discovery/Horizon W (Hologic, Marlborough, Massachusetts) and iDXA/Prodigy (GE Healthcare, Madison, WI) scanners (n=45).

**
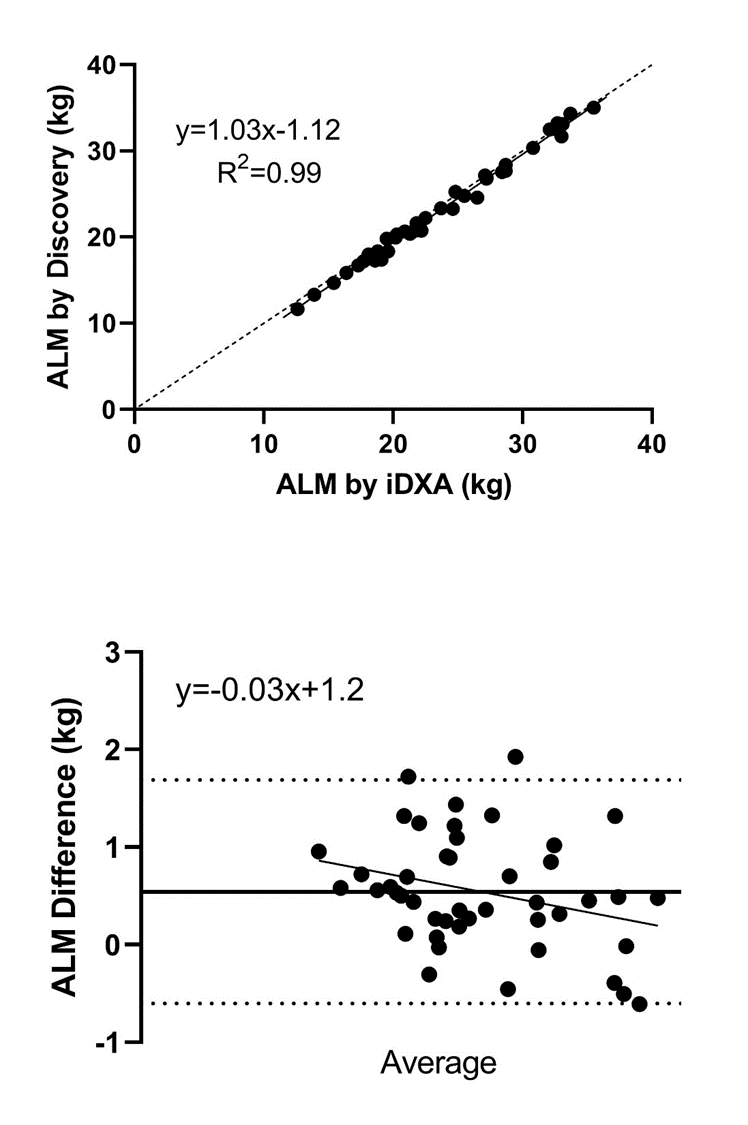
**

| ALM (kg) | Discovery | iDXA | Δ (kg) |
| --- | --- | --- | --- |
| Current Study^†^ | 22.9±6.0 | 23.5±5.8 | 0.54±0.58 |
|  | **Horizon W** | **Prodigy** |  |
| Park et al. | 23.0±4.0 | 24.8±4.3 | 1.79±0.92 |

Results are X±SD. ^†^Bland-Altman, R^2^, 0.09; p<0.05. Mean Δs are both p<0.001.

**Figure 4.** Consort diagram showing participant samples.
